# Deep learning supported mitoses counting on whole slide images: A pilot study for validating breast cancer grading in the clinical workflow

**DOI:** 10.1016/j.jpi.2023.100316

**Published:** 2023-05-04

**Authors:** Stijn A. van Bergeijk, Nikolas Stathonikos, Natalie D. ter Hoeve, Maxime W. Lafarge, Tri Q. Nguyen, Paul J. van Diest, Mitko Veta

**Affiliations:** a Department of Pathology, University Medical Center Utrecht, Postal Box 85500, 3508 GA Utrecht, The Netherlands; b Medical Image Analysis Group (IMAG/e), Eindhoven University of Technology, Eindhoven, The Netherlands; c Computational and Translational Pathology Group, Department of Pathology and Molecular Pathology, University Hospital and University of Zürich, Schmelzbergstrasse 12, 8091 Zurich, Switzerland

**Keywords:** Breast cancer, Artificial intelligence, Digital pathology, Light microscopy, Bloom & Richardson grade, Mitotic index

## Abstract

**Introduction:**

Breast cancer (BC) prognosis is largely influenced by histopathological grade, assessed according to the Nottingham modification of Bloom-Richardson (BR). Mitotic count (MC) is a component of histopathological grading but is prone to subjectivity. This study investigated whether mitoses counting in BC using digital whole slide images (WSI) compares better to light microscopy (LM) when assisted by artificial intelligence (AI), and to which extent differences in digital MC (AI assisted or not) result in BR grade variations.

**Methods:**

Fifty BC patients with paired core biopsies and resections were randomly selected. Component scores for BR grade were extracted from pathology reports. MC was assessed using LM, WSI, and AI. Different modalities (LM-MC, WSI-MC, and AI-MC) were analyzed for correlation with scatterplots and linear regression, and for agreement in final BR with Cohen’s κ.

**Results:**

MC modalities strongly correlated in both biopsies and resections: LM-MC and WSI-MC (R^2^ 0.85 and 0.83, respectively), LM-MC and AI-MC (R^2^ 0.85 and 0.95), and WSI-MC and AI-MC (R^2^ 0.77 and 0.83). Agreement in BR between modalities was high in both biopsies and resections: LM-MC and WSI-MC (κ 0.93 and 0.83, respectively), LM-MC and AI-MC (κ 0.89 and 0.83), and WSI-MC and AI-MC (κ 0.96 and 0.73).

**Conclusion:**

This first validation study shows that WSI-MC may compare better to LM-MC when using AI. Agreement between BR grade based on the different mitoses counting modalities was high. These results suggest that mitoses counting on WSI can well be done, and validate the presented AI algorithm for pathologist supervised use in daily practice. Further research is required to advance our knowledge of AI-MC, but it appears at least non-inferior to LM-MC.

## Highlights

• Breast cancer grading using mitotic count suffers from poor reproducibility.
• Mitotic count on whole slide images correlates well with AI-assisted mitotic count.
• Using AI-assisted mitotic counting can be implemented in daily practice as a viable alternatice to traditional microscope counting.

## Introduction

The yearly worldwide breast cancer (BC) incidence is over 2 million, which makes it the most diagnosed cancer. Female BC currently occupies the fifth place in cancer mortality worldwide, and incidence keeps rising.^1^ However, when diagnosed in an early stage, the prognosis of BC can be good.^1^^,^^2^ One of the strongest factors to determine BC prognosis is histological grade, usually assessed according to the Nottingham modification of Bloom-Richardson (BR) grade.^3^^,^^4^ BR requires the pathologist to score 3 features: tubule formation, nuclear pleomorphism, and mitotic count (MC). Each category gets a score from 1 to 3. Scores 3–5 define grade 1, 6–7 grade 2, and 8–9 make up grade 3 BC. Grade 1 cancers have a significantly better survival than grade 2 or 3 cancers.^3^^,^^5^^,^^6^ Studies have shown histological grading, tumor size, and lymph node status to be of equal importance for the prognosis of BC.^5^^,^^6^ Furthermore, histological grade proved to be decisive in up to a third of treatment decisions.^7^

MC is, as a marker of tumor proliferation, the strongest constituent of BR grade, and a high MC is associated with poor prognosis.^8^, ^9^, ^10^ Several studies have shown a moderate to good reproducibility for BR.^11^, ^12^, ^13^ When focusing solely on MC, reproducibility also ranges from moderate to high.^14^^,^^15^ However, concerns for reproducibility still exist as 1 recent study again found substantial inter- and intra-laboratory variations in BR in more than 33 000 patients.^7^ Because of these variations and the importance of MC for the prognosis of BC, higher reproducibility is required.

With the development of digital whole slide imaging (WSI), breast cancer diagnostics have increasingly been performed digitally as WSI have been validated for diagnostic purposes.^16^^,^^17^ It has been argued that standard WSI has limitations for reliable histologic grading, as the quality of the images may not be high enough for properly assessing the MC in all cases due to lack of a z-axis (i.e. fine-tuning of the focal length), which pathologists often use when microscopically assessing MC. Pathologist familiarity with WSI in the clinical workflow might also be limiting factor. Also, a change in ergonomics is required when using a computer mouse instead of a microscope which might further influence pathologist opinion on WSI. Two studies have shown that MCs in WSI and traditional light microscopy (LM) show comparable results.^18^^,^^19^ However, other studies suggest that although the inter-observer agreement on WSI is similar to LM, MC tends to be systematically lower on WSI.^16^^,^^17^^,^^20^^,^^21^

The increased usage of WSI has stimulated the rise of artificial intelligence (AI) algorithms in pathology. Several of these have been developed for assisting the pathologist in performing MC, expecting to improve the reproducibility of MC, often tested in validation cohorts.^5^^,^^19^^,^^22^, ^23^, ^24^, ^25^, ^26^, ^27^ The next step is to test AI algorithms in a clinical setting. The present study validates an in-house developed AI algorithm for mitoses counting in BC on digital WSI by comparing AI supported MC to light microscopic MC and evaluating influence of putative differences of these MC modalities on BR grade in breast cancer.

## Methods

### Study design and population

Fifty BC patients with paired core biopsies and resections were randomly selected from the workflow of the Department of Pathology at the UMC Utrecht between December 2018 and February 2020. For each patient, tubular differentiation (scored 1, 2, or 3) and nuclear polymorphism scores (1, 2, or 3) according to Elston and Ellis^3^ were taken from the original pathology report (14 grade 1, 28 grade 2, and 8 grade 3). An approval from our Institutional Review Board was requested and granted under the application number TCBio-20-777.

An experienced Pathologist Assistant (PA) trained in breast microscopy first determined the most cellular and proliferative area of the tumor using LM without prior knowledge of the BR grade and MC. The MC was reassessed using LM (LM-MC) in 2 mm^2^ of adjacent fields.^14^ After getting the exact count, MC was scored as 1, 2, or 3 points, for respectively ≤7, 8–12, and ≥13 mitoses. After a washout period of at least 2 months, MC was assessed digitally using WSI (WSI-MC), and after another 2 months washout period, MC was assessed supported by the AI algorithm (AI-MC).

Prior to start using the AI algorithm, a standard operation procedure document (SOP) was made for the AI tool and the PA was trained on the usage of the tool on the test PACS environment.

### Digital pathology and AI

Slides had routinely been scanned within the workflow of the UMC Utrecht at 40× magnification (resolution of 0.22 μm per pixel) with a Nanozoomer 2.0-XR (Hamamatsu, Japan). All WSI were viewed using standard high-resolution 4k computer screens in the Sectra PACS (Linköping, Sweden).

The automated mitosis detection system was developed internally based on the methodology introduced by Cireşan et al^28^ and the improvements upon this work by Lafarge et al.^29^ The model was trained using Tensorflow 1.12 on python 2.7 and is based on rotation invariant group convolutional neural networks. We used the TUPAC16 and AMIDA13 grand challenge (GC) dataset to train the network as well as a smaller annotated dataset containing mostly hard-negatives and ink artifacts to improve robustness. Most GC datasets include examples from within the tumor and rarely from the periphery of the slide—here the most ink artifacts and other mimics are found—which can lead to performance degradation when whole slide inference is performed.

The model is a 6-layer group CNN, the architecture is extensively described in Lafarge et al.^29^ In short, we used a patch size of 68×68 pixels with a batch size of 64 and it was trained on NVIDIA K80 and NVIDIA V100 hardware. We evaluated the performance of the model on test sets of the GC datasets and used the F2-score threshold for the clinical implementation. The F2-score threshold gives more weight to recall than precision in contrast to F1-score which gives equal weight to both. This threshold allows the pathologist to review more objects while not overwhelming them with too many objects to review.

**Fig. 1:**
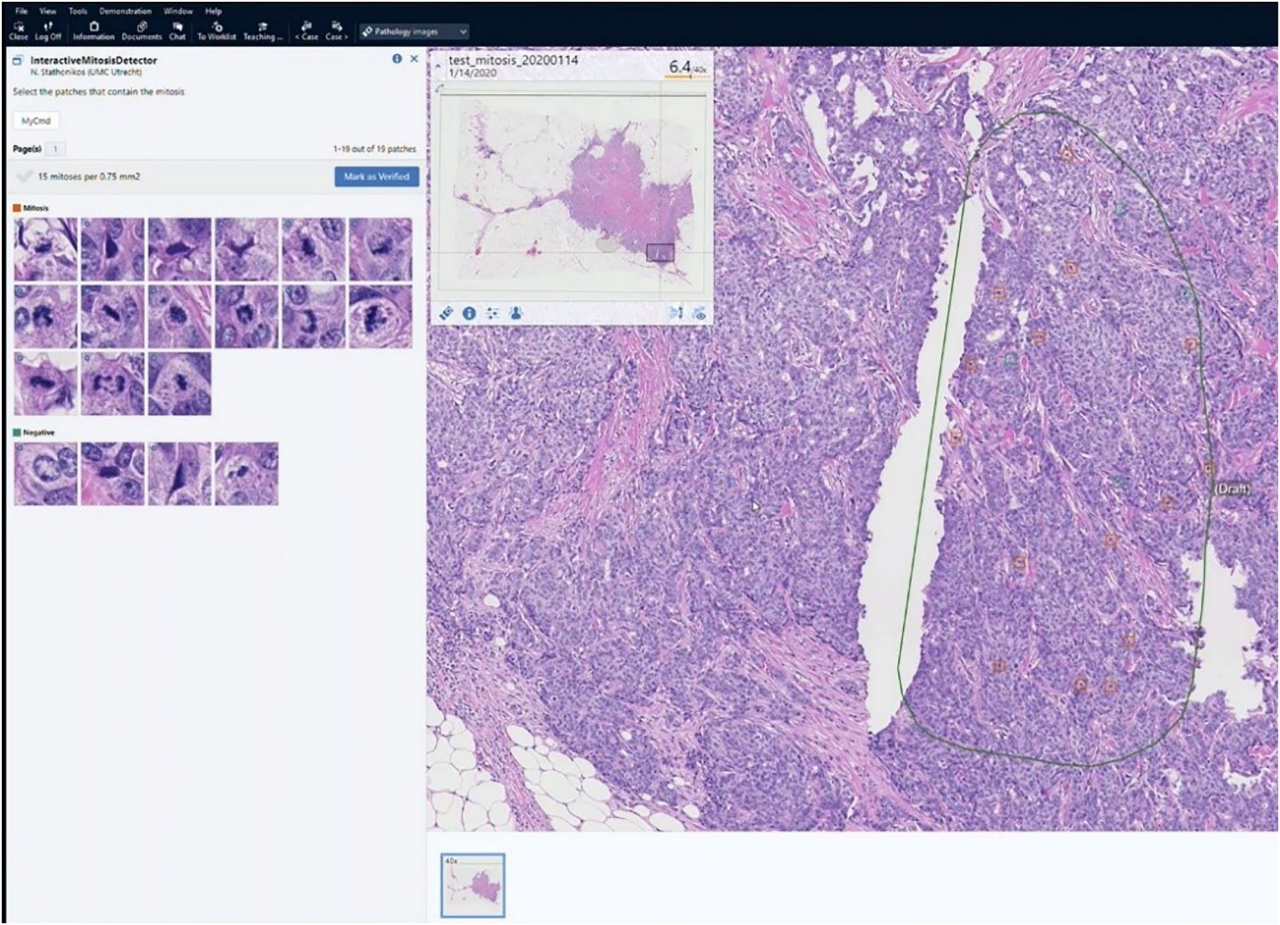
Screenshot of the Sectra PACS where an area of interest has interactively been drawn on the right-hand side, after which an AI algorithm has found candidate mitoses and mitosis-like objects, which are displayed in the galleries in the upper left-hand side of the screen. By clicking on a thumbnail in either of the galleries, the PACS displays the candidate object in the center on the right for review, and false positives can be dragged to the negative gallery and vice versa, after which a final AI supported MC is established.

The model takes large image patch of 40× resolution and generates a probability map of that patch. Then by using local-maxima extraction, it gets the positions of mitosis on that patch. The MC AI algorithm (both model and integration with PACS) was in-house developed. In the Sectra PACS, an area of interest of the appropriate size of 2 mm^2^ (as described for LM-MC) is interactively drawn, after which the algorithm automatically identifies candidate mitoses and mitoses-like objects and displays them in 2 galleries. Objects are interactively reviewed and dragged to the correct gallery, resulting in a final AI MC per 2 mm^2^ (Fig. 1).

### Data analysis

Using the MC from the 3 modalities, 3 BR grades were composed for each biopsy and resection as usual by summing up the scores from tubular differentiation, nuclear polymorphism, and MC, total score 3–5 defining grade 1, scores 6–7 grade 2, and scores 8–9 grade 3. Data for biopsies and resections were separately analyzed. MC data were pairwise displayed in logarithmic scatterplots with reference lines between the different MC modalities and R^2^ was calculated to detect systematic differences. To assess the concordance in BR resulting from the different MC modalities, crosstabs were created, using Cohen’s κ to assess BR agreement between the different MC modalities.^30^ Scores of 0 meant no agreement, 0.01–0.20 none to slight, 0.21–0.40 fair, 0.41–0.60 moderate, 0.61–0.8 substantial, and 0.81–1.00 almost perfect agreement.^32^ All statistics were done using Python version 3.8.5. and scikit-learn 1.0.2 and pingouin 0.5.2 python packages.

## Results

### Biopsies

**Fig. 2:**
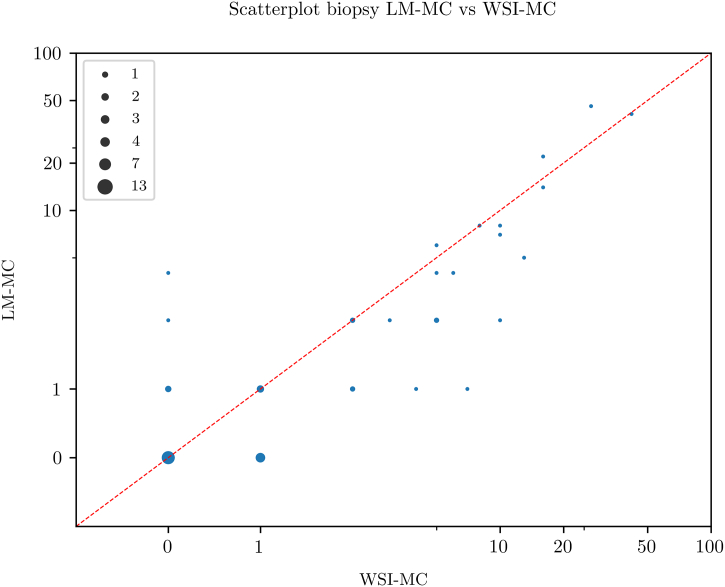
Scatterplot showing a high concordance between whole slide image-based digital mitotic count (WSI-MC) and light microscopic MC (LM-MC) in 50 breast cancer biopsies.

**Fig. 3:**
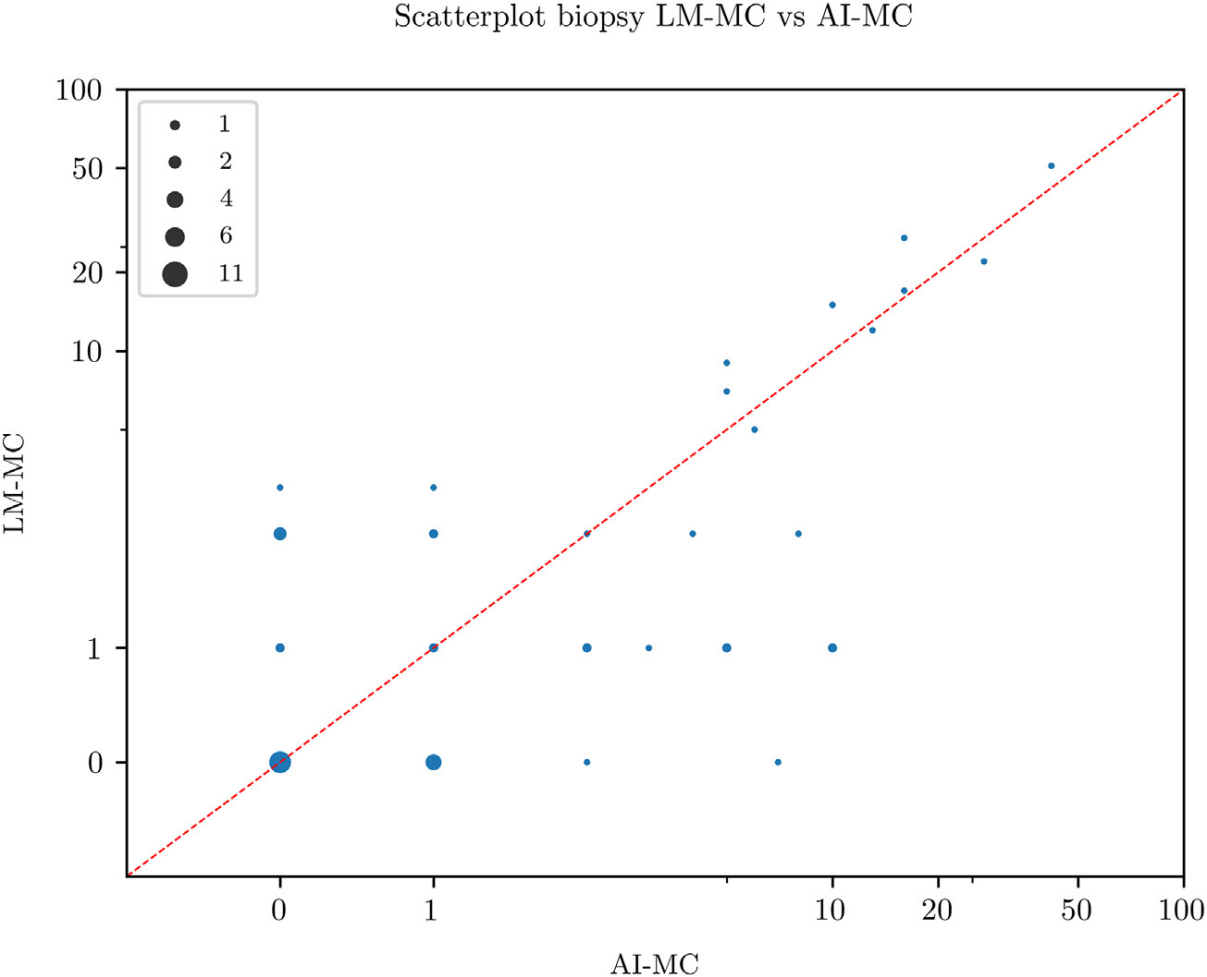
Scatterplot showing a high concordance between artificial intelligence-based mitotic count (AI-MC) and light microscopic MC (LM-MC) in 50 breast cancer biopsies.

**Fig. 4:**
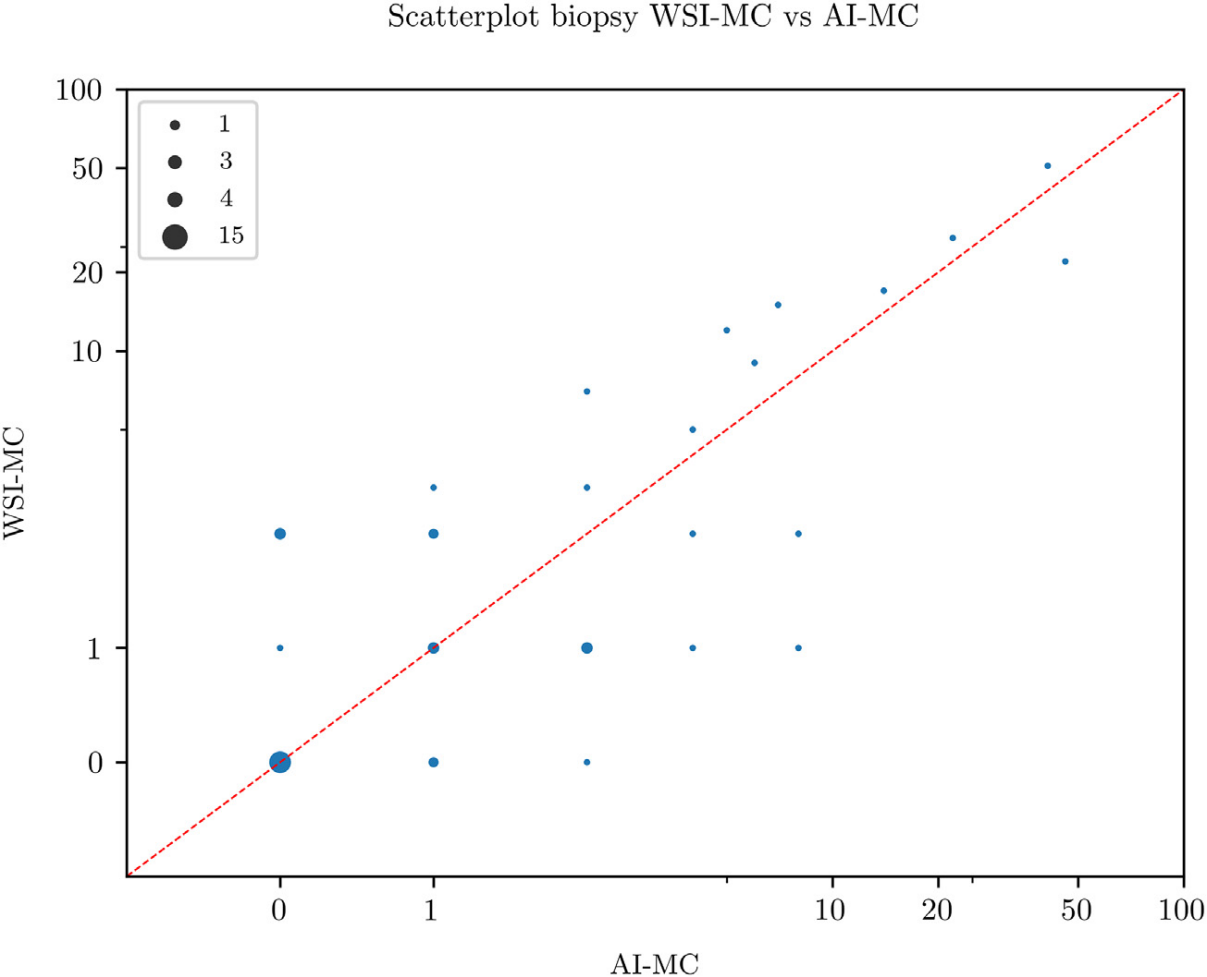
Scatterplot showing a high concordance between artificial intelligence-based mitotic count (AI-MC) and whole slide image-based digital MC (WSI-MC) in 50 breast cancer biopsies.

Scatterplots for pairwise comparison between the 3 MC modalities are shown in Fig. 2, Fig. 3, Fig. 4. All MC modalities were strongly correlated: R^2^ between LM-MC and WSI-MC was 0.85, 0.85 between LM-MC and AI-MC, and 0.77 between WSI-MC and AI-MC.

The crosstabs for the BR grades resulting from the different MC modalities are shown in Table 1, Table 2, Table 3, all showing high κ values: 0.93 for LM-MC versus WSI-MC-based BR, 0.89 for LM-MC versus AI-MC-based BR, and 0.96 for WSI-MC versus AI-MC-based BR.

**Table 1:** Crosstab between Bloom & Richardson (BR) grade based on light microscopic mitotic count (LM-MC) and artificial intelligence supported MC (AI-MC) in 50 breast cancer biopsies (κ=0.894, 95% CI 0.78–1.01).

	AI-MC-based grade			
LM-MC-based grade	1	2	3	Total
1	17	0	0	17
2	1	26	1	28
3	0	1	4	5
Total	18	27	5	50

**Table 2:** Crosstab between Bloom & Richardson (BR) grade based on light microscopic mitotic count (LM-MC) and whole slide image-based digital MC (WSI-MC) in 50 breast cancer biopsies (κ=0.928, 95% CI 0.83–1.01).

	WSI-MC-based grade			
LM-MC-based grade	1	2	3	Total
1	17	0	0	17
2	1	27	0	28
3	0	1	4	5
Total	18	28	4	50

**Table 3:** Crosstab between Bloom & Richardson (BR) grade based on whole slide image-based digital mitotic count (WSI-MC) and artificial intelligence supported MC (AI-MC) in 50 breast cancer biopsies (κ=0.964, 95% CI 0.90–1.03).

	AI-MC-based grade			
WSI-MC-based grade	1	2	3	Total
1	17	0	0	17
2	1	26	1	28
3	0	1	4	5
Total	18	27	5	50

### Resections

**Fig. 5:**
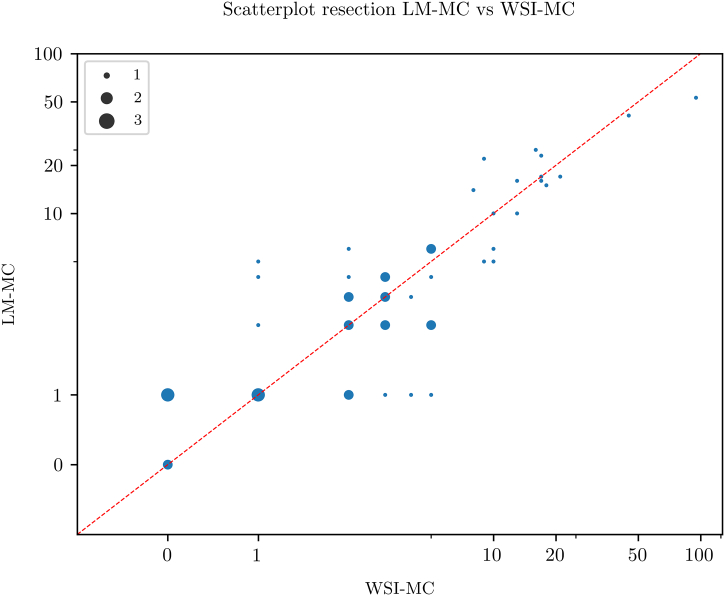
Scatterplot showing a high concordance between whole slide image-based digital mitotic count (WSI-MC) and light microscopic MC (LM-MC) in 50 breast cancer resections.

**Fig. 6:**
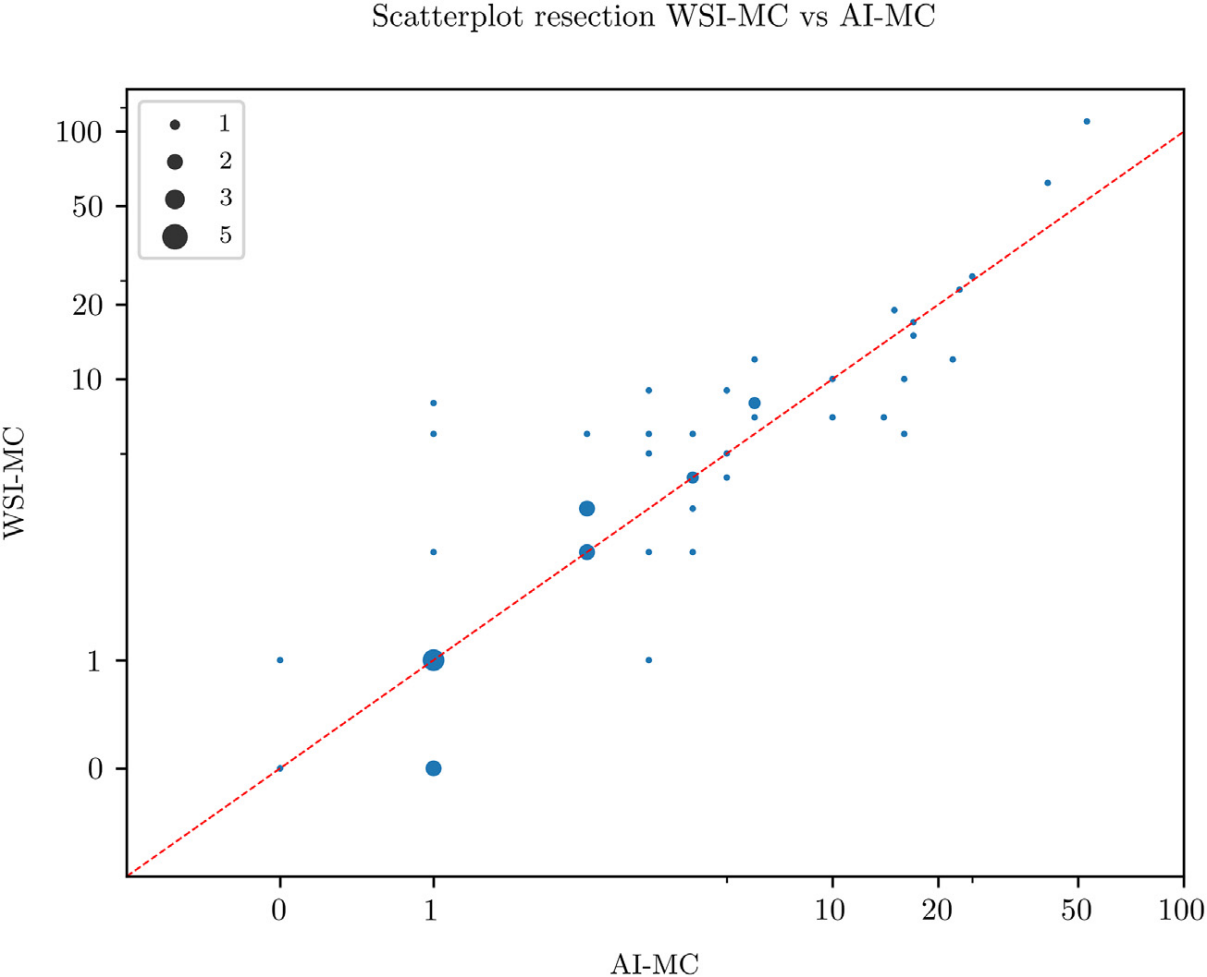
Scatterplot showing a high concordance between artificial intelligence-based mitotic count (AI-MC) and light microscopic MC (LM-MC) in 50 breast cancer resections.

**Fig. 7:**
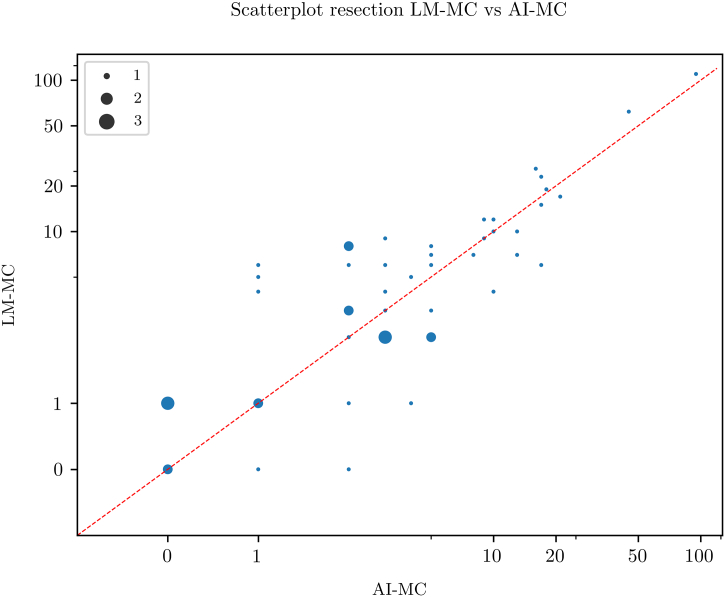
Scatterplot showing a high concordance between artificial intelligence-based mitotic count (AI-MC) and whole slide image-based digital MC (WSI-MC) in 50 breast cancer resections.

Scatterplots for pairwise comparison between the 3 MC modalities are shown in Fig. 5, Fig. 6, Fig. 7. All MC modalities were strongly correlated: R^2^ between LM-MC and WSI-MC was 0.83, 0.95 between LM-MC and AI-MC and 0.83 between WSI-MC and AI-MC.

The crosstabs for the BR grades resulting from the different MC modalities are shown in Table 4, Table 5, Table 6, all showing high κ values: 0.83 for LM-MC-based BR versus WSI-MC, 0.83 for LM-MC versus AI-MC-based BR, and 0.73 for WSI-MC versus AI-MC-based BR.

**Table 4:** Crosstab between Bloom & Richardson (BR) grade based on light microscopic mitotic count (LM-MC) and whole slide image-based digital MC (WSI-MC) in 50 breast cancer resections (κ=0.834, 95% CI 0.70–0.97).

	WSI-MC-based grade			
LM-MC-based grade	1	2	3	Total
1	13	1	0	14
2	2	24	2	28
3	0	0	8	8
Total	15	25	10	50

**Table 5:** Crosstab between Bloom & Richardson (BR) grade based on light microscopic mitotic count (LM-MC) and artificial intelligence supported MC (AI-MC) in 50 breast cancer resections (κ=0.825, 95% CI 0.68–0.97).

	AI-MC-based grade			
LM-MC-based grade	1	2	3	Total
1	13	1	0	14
2	2	26	0	28
3	0	2	6	8
Total	15	29	6	50

**Table 6:** Crosstab between Bloom & Richardson (BR) grade based on whole slide image-based digital mitotic count (WSI-MC) and artificial intelligence supported MC (AI-MC) in 50 breast cancer resections (κ=0.732, 95% CI 0.56–0.90).

	AI-MC-based grade			
WSI-MC-based grade	1	2	3	Total
1	13	2	0	15
2	2	23	0	25
3	0	4	6	10
Total	15	29	6	50

## Discussion

In this study, we investigated whether mitoses counting in BC using digital WSI compares better to LM-MC when assisted by AI, and to which extent differences in digital MC (AI assisted or not) result in BR grade variations.

For biopsies, LM-MC and AI-MC showed an equal R^2^ when compared with LM-MC and WSI-MC, despite the latter already correlating well. For resections, R^2^ of LM-MC and AI-MC even surpassed that of LM-MC and WSI-MC. This data suggests that not only does AI correlate as well as WSI with LM for mitotic count, but also might perhaps compare better to LM.

It was noted that AI-MC resulted in systematically slightly lower MC values compared to LM-MC and WSI-MC. This indicates that the AI algorithm may miss some mitoses and needs further improvement. However, as the observer checked the results, the observer may not have been critical enough when reviewing mitoses which the AI classified as mitoses-like objects. This could lower AI-MC compared to LM-MC and WSI-MC and underlines the importance of careful human supervision of the output of algorithms when AI is used in daily practice.

Several other studies showed similar results regarding the comparability between LM-MC and WSI-MC.^16^^,^^20^^,^^31^^,^^32^ Noted differences between LM-MC and WSI-MC were perceived to be within the range of inter-observer differences in LM-MC. Also, studies which used 40× magnification for scanning and high-resolution displays noted that differences between WSI and LM tended to get smaller, suggesting that a certain standard of technology is required for proper mitoses counting on WSI. As to AI, a recent study applying AI to select a mitoses hotspot in which to count showed improved inter-observer agreement in interactive mitoses counting on WSI, with similar inter-observer κ values for LM-MC and AI-MC.^19^ However, one study demonstrated higher inter-observer agreement for AI-MC compared to LM-MC, and a substantial saving in time.^33^ So, different studies seem to point at least to non-inferiority of AI-MC compared to LM-MC in BC. The potential to save time is another reason to further explore the possibilities of AI.

Both biopsies and resections showed near perfect agreement in BR between different modalities, although the κ for WSI-MC versus AI-MC-based BR in the resection group was slightly lower. This indicates that differences in MC between different modalities hardly influence BR grade.

One study compared BR based on LM and WSI in over 1600 cases, showing a strong association (Cramer’s V: 0.58) between both modalities.^16^ Another study focusing on inter-observer differences in BR when using WSI, showed the concordance to be similar to inter-observer differences in BR using LM.^21^ These studies substantiate our results. To the best of our knowledge, no previous study has been conducted that compares agreement of BR using LM-MC or WSI-MC and AI-MC. The high agreement in BR in this study is probably related to 2 factors. Firstly, WSI-MC and AI-MC were performed on the exact same slide as LM-MC, whereas larger tumors may be heterogeneous across different tissue blocks. Secondly, grading in different modalities was assessed by the same observer, causing the criteria for mitotic figures to be interpreted singularly and increasing the chance of selecting the same hotspot.

This study has some limitations. First, the gold-standard is LM-MC assessed by a single observer. Due to significant inter-observer differences for LM-MC, a study with multiple observers may provide a more realistic view on the added value of AI. Another option would be to use Phosphohistone H3 immunohistochemistry, which enhances recognition of mitotic figures and may make LM-MC (and perhaps even AI-MC) more reproducible.^34^ Secondly, this study has a relatively small number of cases.

In daily pathology practice, digital WSI is increasingly used worldwide. This study, in combination with previous studies in this field, shows WSI-MC to be suitable for grading BC. Especially pathology laboratories which have a digital workflow could thereby incorporate WSI-MC in their daily practice of grading BC.

In general, AI algorithms show great promise in improving pathology practice. This study demonstrates that mitoses counting in BC can not only be performed by an AI algorithm, but also might compare better to LM than WSI. We expect the next generation algorithms to be improved even further.^35^ These algorithms may also save valuable interaction time for the pathologist, especially when algorithms run in the background on WSI, providing the pathologist with mitotic hotspots.

In conclusion, this first validation study shows that WSI-MC might compare better to LM-MC by using AI. Agreement between different modalities for BR was high. WSI-MC appears as a viable alternative to LM-MC. Further research is required to advance our knowledge of AI-MC, but it appears at least non-inferior to LM-MC and has the potential to save time.

## Funding sources

This research did not receive any specific grant from funding agencies in the public, commercial, or not-for-profit sectors.

## Conflict of interest

The authors declare that they have no known competing financial interests or personal relationships that could have appeared to influence the work reported in this paper.
